# Localized Delivery of Cl-Amidine From Electrospun Polydioxanone Templates to Regulate Acute Neutrophil NETosis: A Preliminary Evaluation of the PAD4 Inhibitor for Tissue Engineering

**DOI:** 10.3389/fphar.2018.00289

**Published:** 2018-03-28

**Authors:** Allison E. Fetz, Indira Neeli, Karyl K. Buddington, Robert W. Read, Matthew P. Smeltzer, Marko Z. Radic, Gary L. Bowlin

**Affiliations:** ^1^Department of Biomedical Engineering, University of Memphis, Memphis, TN, United States; ^2^Department of Microbiology, Immunology and Biochemistry, University of Tennessee Health Science Center, Memphis, TN, United States; ^3^Animal Care Facilities, University of Memphis, Memphis, TN, United States; ^4^TriMetis Life Sciences, Memphis, TN, United States; ^5^Division of Epidemiology, Biostatistics, and Environmental Health, School of Public Health, University of Memphis, Memphis, TN, United States

**Keywords:** electrospinning, tissue regeneration, biomaterials, neutrophils, neutrophil extracellular traps, NETosis, Cl-amidine

## Abstract

Upon interaction, neutrophils can potentially release neutrophil extracellular traps (NETs) on the surface of an implanted electrospun template, which may be a significant preconditioning event for implantable biomaterials of yet unknown consequences. In this study, we investigated the potential of polydioxanone templates as a delivery vehicle for Cl-amidine, an inhibitor of peptidyl arginase deiminase 4 (PAD4), and if drug elution could attenuate PAD4-mediated NETosis in the vicinity of implanted templates. Electrospun polydioxanone templates were fabricated with distinct architectures, small diameter (0.4 μm) or large diameter (1.8 μm) fibers, and incorporated with 0–5 mg/mL Cl-amidine to examine dose-dependent effects. Acute neutrophil-template interactions were evaluated *in vitro* with freshly isolated human neutrophils and *in vivo* with a rat subcutaneous implant model. The *in vitro* results suggest large diameter templates with 0 mg/mL Cl-amidine significantly attenuate NETosis compared to small diameter templates. As the drug concentration increased, NETosis was significantly decreased on small diameter templates in a dose-dependent manner. The opposite was observed for large diameter templates, indicating multiple mechanisms of NETosis may be regulating neutrophil template preconditioning. Similar results were observed *in vivo*, verifying local NETosis inhibition by Cl-amidine eluting templates in a physiological environment. Importantly, large diameter templates with Cl-amidine enhanced neutrophil invasion and survival, supporting the potential for long-term modulation of tissue integration and regeneration. This preliminary study demonstrates a novel delivery vehicle for Cl-amidine that can be used to regulate acute NETosis as the potential critical link between the innate immune response, inflammation, and template-guided tissue regeneration.

## Introduction

When a biomaterial template is implanted in the body, a highly orchestrated series of events is triggered that ideally harnesses the body as a bioreactor, resulting in *in situ* tissue regeneration. Almost instantly, soluble blood serum proteins adsorb on the surface of the biomaterial. The neutrophils swarming into the local microenvironment around the template interact with these proteins and other cells to begin orchestrating the complex responses leading to tissue integration, repair, and regeneration or fibrosis. Regulating the body's innate immune response to an acellular template to harness it as a bioreactor to promote *in situ* tissue regeneration is an emerging focus of tissue engineering. Instead of designing inert materials, tissue engineers aim to develop temporary templates that prevent fibrosis while dynamically guiding tissue integration and regeneration, driven by the innate immune system.

In terms of biomaterial-guided *in situ* tissue regeneration, the critical link between the innate immune response and the healing outcome may be the neutrophil. Historically, neutrophils have been regarded as the rapidly cleared, suicidal killers of the innate immune response. They functioned to simply clean up the microenvironment around a biomaterial before undergoing apoptosis and swift removal by macrophages. However, insightful research in the last decade suggests that the neutrophil is capable of much more than previously understood with regards to biomaterial-guided tissue regeneration (Selders et al., 2017).

Neutrophils adopt distinct phenotypes, similar to macrophages, which are influenced by their microenvironment (Fridlender et al., 2009). They release signals through degradation of the extracellular matrix and rapidly secrete pre-packaged granules that can modulate their phenotype and influence the recruitment of additional cells (Cowland and Borregaard, 2016; de Oliveira et al., 2016). Additionally, neutrophils are able to survive significantly longer after extravasation than once accepted (Tak et al., 2013), suggesting their ability to synthesize and secrete factors for early-stage immune system modulation that may have long-term effects. Finally, several recent studies have identified that neutrophils can actually reverse migrate from an area of inflammation, relocating to new areas of the body to potentially educate the immune system (Elks et al., 2011; Wang et al., 2017).

Importantly, neutrophils are able to expel their DNA along with granular contents to form neutrophil extracellular traps (NETs) through a specialized form of antimicrobial cell death called NETosis (Brinkmann et al., 2004). During NETosis, chromatin within the nucleus decondenses while the nuclear envelop and granule membranes disintegrate, resulting in a mixing of the potent granule cargo and DNA. Eventually, a cloud-like, fibrillary network of DNA that is decorated with antimicrobial proteins, proteases, and histones is extruded into the microenvironment. Importantly, NETing neutrophils are protected from pre-emptive clearing by macrophages, resulting in NETs that are difficult to contain (Branzk et al., 2014).

During NETosis, chromatin decondensation occurs with deimination of histone H3 to citrullinated histone H3 (CitH3) by the enzyme peptidyl arginase deiminase 4 (PAD4) (Neeli et al., 2008; Wang et al., 2009). The basic amino acid residue, arginine, is converted to a neutral citrulline residue, which weakens the interaction between the histone and chromatin, leading to an unraveling of chromatin. When the activity of PAD4 is blocked with an inhibitor or eliminated in a mouse knockout model, the production of NETs is severely reduced for antibacterial immunity and in pathological deep vein thrombosis (Li et al., 2010; Martinod et al., 2013; Lewis et al., 2015).

While functioning as a classic antimicrobial response, NETosis has also been implicated in various forms of tissue fibrosis, thus making it a prominent therapeutic target. In particular, neutrophils have been linked to pulmonary fibrosis, fibrosis after myocardial infarction, and atherosclerosis (Jones et al., 1998; Rainer et al., 2014; Marino et al., 2015). Dysregulated release of NETs appears to contribute to the overproduction of dense fibrotic matrix in tissue fibrosis. In terms of tissue regeneration, our group has recently demonstrated that the excessive presence of NETs inhibits tissue integration with an electrospun biomaterial and may promote fibrotic encapsulation (Fetz et al., 2017). In the study, we electrospun polydioxanone (PDO), a synthetic resorbable biomaterial, to create two distinct material architectures, templates with small diameter (SD, 0.3 ± 0.1 μm) or large diameter (LD, 1.9 ± 1.0 μm) fibers and pores. Using fresh human peripheral blood neutrophils *in vitro* and a subcutaneous rat implant model, we showed that the microenvironment created by the template architecture differentially modulates NETosis and formation of fibrotic tissue *in vivo*, respectively.

In the present work, we confirmed the experiments of our previous study and extended them by incorporating varying concentrations of Cl-amidine (Cl-Am), a haloacetamidine-based compound that irreversibly inhibits (PAD4) (Luo et al., 2006), into the electrospun templates. Cl-Am covalently modifies the cysteine residue in the active site that is critical for PAD4 enzymatic activity and functions in a dose-dependent manner (Willis et al., 2011). The purpose of this study was to incorporate Cl-Am into electrospun PDO templates for local delivery, measure its activity, and further assess the potential of the drug elution to inhibit PAD4-mediated citrullination of histone H3 and reduce template-induced NETosis. We hypothesized that elution of Cl-Am from the templates would attenuate NETosis with the greatest effect seen on SD templates. SD and LD templates were fabricated with 0-5 mg/mL Cl-Am, and NETosis was evaluated in response to the templates *in vitro* with freshly isolated, human peripheral blood neutrophils and *in vivo* with a subcutaneous implant model in a rat. The results were analyzed based on the temporal degree of NETosis in response to the template architectures and Cl-Am concentrations. Ultimately, regulating the release of template-preconditioning NETs from the interacting neutrophils may modulate the early-stage innate immune response, promoting or even enhancing guided tissue regeneration.

## Materials and methods

### Activity of Cl-Am after solvent exposure

Cl-Am (trifluoroacetate salt) (Cayman Chemical, Item No. 10599, Batch No. 0468497-71) activity was quantified by a PAD4 Inhibitor Screening Assay (Cayman Chemical, Item No. 700560) following manufacturer protocol to evaluate activity after exposure to the organic electrospinning solvent, 1,1,1,3,3,3-hexafluoro-2-propanol (HFP, Oakwood Products, Inc.), simulating processing. Cl-Am was suspended in HFP, and 1x Hank's buffered salt solution (HBSS) to result in final assay concentrations of 147.8, 29.5, 5.9, 1.18, 0.24, 0.047, and 0 μM. 40 μL of each HFP sample (*n* = 4) were pipetted into the wells of a 96-well culture plate, and the plate was placed in fume hood to allow evaporation of HFP overnight at room temperature. Then, the drug was resuspended in 40 μL of HBSS for detection. 5 μL of the HBSS and resuspended HFP samples were assayed according to manufacturer protocol and were read on a SpectraMax i3x Multi-Mode Microplate Reader (excitation 410 nm and emission 475 nm) to determine fluorescent intensity. After taking the log transform of molarity, the average fluorescent intensity and standard deviation for each sample were plotted against Cl-Am concentration.

### Template fabrication and characterization

PDO (Sigma Aldrich Co.) was dissolved overnight in HFP at varying concentrations (Table 1) ranging from 65 to 100 and 170 to 200 mg/mL to create SD and LD templates, respectively. 0, 1, 2.5, or 5 mg/mL of Cl-Am was added to the polymer solution and dissolved for 2 h with gentle agitation before electrospinning. Solutions were loaded into a syringe with an 18-gauge blunt needle tip attached to the positive lead of a power source (Spellman CZE1000R, Spellman High Voltage Electronics Corp.) and placed on a syringe pump (Model No. 78-01001, Fisher Scientific). Solutions were electrospun with optimized flow rate, airgap distance, and applied voltage (Table 1). Fibers were collected on a grounded, stainless steel rectangular mandrel (200 × 750 × 5 mm) that was rotating at 1,250 rpm and translating 6.5 cm/s over 13 cm. The templates (thickness 0.10–0.25 mm) were stored at −20°C until analyses.

**Table 1:** Electrospun templates were fabricated with optimized parameters.

	**Polymer concentration [mg/mL]**	**Cl-Am concentration [mg/mL]**	**Flow rate [mL/h]**	**Airgap distance [cm]**	**Applied voltage [+kV]**
SD	65	0	2	13	22
	100	1	2.5	15	18
	100	2.5	2.5	15	18
	100	5	2.5	15	18
LD	170	0	6	13	22
	200	1	3	18	28
	200	2.5	3	18	28
	200	5	3	18	28

*Polymer and Cl-Am concentration were varied to create two distinct groups of SD and LD templates*.

For scanning electron microscopy, templates were coated with 5 nm of gold-palladium by sputter coating in an argon gas field. The materials were then imaged with a FEI Nova NanoSEM 650 with field emission gun at + 20 kV with a working distance of 5 mm to generate scanning electron micrographs (SEMs). Fiber diameter and pore diameter were characterized by analyzing the SEMs in FibraQuant 1.3 software (nanoTemplate Technologies, LLC). Average fiber and pore diameters with corresponding standard deviations were determined from a minimum of 250 semi-automated random measurements per image and 60 random manual measurements per image, respectively.

### Cl-Am template elution

Electrospun templates were characterized for the delivery of Cl-Am into supernatant over 5 days. 10 mm diameter discs of the templates (*n* = 6) were placed in a 48-well culture plate, and 300 μL of HBSS were added to each well. After incubation at 37°C for 15, 30, 45 min, 1, 2, 3, 6, 12 h, 1, 2, 3, 4, and 5 days, the supernatant was removed and refreshed with 300 uL of HBSS. The collected supernatant was frozen and stored at −20°C until analysis. Activity of Cl-Am was detected using the PAD4 Inhibitor Screening Assay following manufacturer protocol, and the resulting fluorescent intensities were used to determine Cl-Am concentration within the samples. An average and standard deviation were determined for each template type.

### *In vitro* evaluation with fresh human peripheral blood neutrophils

Neutrophils were isolated from blood obtained from healthy donors from Tennessee Blood Services in accordance with protocols approved by the University of Tennessee Institutional Review Board as previously described (Fetz et al., 2017). Informed and written consent was obtained by Tennessee Blood Services. After isolation, neutrophils were resuspended at a density of 2.4 × 10^6^ cells/mL in HBSS without calcium and magnesium and with sodium bicarbonate, 10 mM HEPES, and 0.2% autologous serum. 10 mm diameter discs of the PDO templates (*n* = 4) were disinfected by utraviolet (UV) irradiation with a Spectroline handheld lamp (8 watt, Part No. EN280L) for 10 min per side in a sterile laminar flow hood with 365 nm wavelength at a working distance of 9.5 cm. The templates were placed in a 48-well culture plate and hydrated with 50 μL of sterile HBSS. Immediately following hydration, 250 μL of the cell suspension (600,000 cells) were pipetted onto the templates, and the templates were cultured at 37°C and 5% CO_2_. After 3 and 6 h, the plates were placed on ice, inhibiting further stimulation of the neutrophils. Supernatant was carefully removed, and the cells were fixed in the wells with 10% neutral buffered formalin. Cellularized templates were stored in 10% formalin at 4°C until analysis.

#### Fluorescence quantification of NETs

Fluorescence microscopy of the templates (*n* = 4) was used to quantify the degree of NETosis as previously described (Fetz et al., 2017). Briefly, templates were stained with 100 nM SYTOX green (SG) extracellular DNA stain (Life Technologies, Cat. No. S7020) in deionized water for extruded NETs followed by 4′,6-diamidino-2-phenylindole (DAPI) fixed cell nuclei stain (Life Technologies, Cat. No. R37606) at stock concentration for intact nuclei. To determine the relative degree of NETosis, the ratio of blue to green staining (BG), representing the area of intact cell nuclei to extruded NETs, within an image was quantified using MATLAB Release 2012a. The BG ratios were normalized to cell number within an image, and an average and standard deviation were calculated for each template type.

#### Infrared scanning and quantification of CitH3

An On-cell Western assay was performed to quantify template-bound CitH3 as described (Fetz et al., 2017). Briefly, human histone H3 (citrulline R2 + R8 + R17) peptide (Abcam, Product code ab32876) was spotted on an Immobilon-FL PVDF membrane and dried overnight to create a standard curve. The standard curve and cellularized templates (*n* = 4) were blocked with 5% non-fat milk for 1 h at room temperature, then incubated with rabbit anti-human histone H3 (citrulline R2 + R8 + R17) antibody (Abcam, Product code ab5103) at a 1:200 dilution in 5% milk overnight at 4°C. After incubation with primary antibody, three washes were performed with 0.1% Tween-20 in HBSS for 5 min at room temperature with gentle agitation. Following, the standard curve and templates were incubated with IRDye 800CW donkey anti-rabbit (LICOR, Part no. 926-32213) at a 1:20,000 dilution in 5% milk with 0.1% Tween-20 and 0.01% SDS for 1 h at room temperature. Non-cellularized templates were incubated with secondary antibody only, serving as negative controls. After two washes with 0.1% Tween-20 in HBSS and one final wash with HBSS, the standard curve and templates were scanned on the 800 nm channel of the Odyssey CLx Infrared Imaging System (LICOR) to generate full-thickness template fluorescence. The scans were analyzed with Image Studio Version 5.x (LICOR).

For the standard curve, relative fluorescent intensities were acquired by placing circular markers over the area of a spot. The average and standard deviations were plotted against the mass of CitH3 to create a standard curve. For the templates, the relative fluorescent intensities were measured by encompassing the area of the sample and extrapolating it to the area for a 10 mm diameter disc, which was normalized to template thickness. The final relative fluorescent intensity was converted to mass of CitH3 using the standard curve, and an average and standard deviation were generated for each template type.

### *In vivo* rat subcutaneous implant model

**Figure 1:**
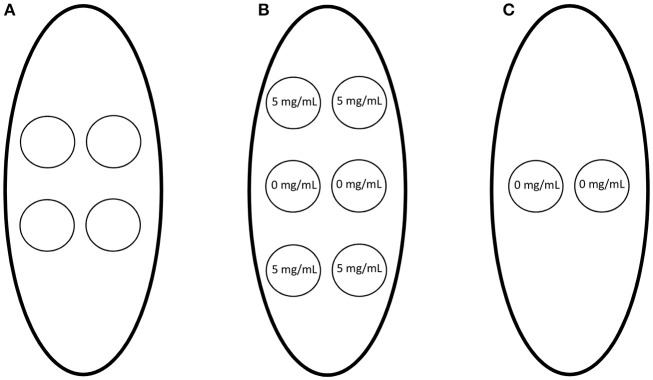
Electrospun templates with 0–5 mg/mL Cl-Am were implanted subcutaneously on the dorsa of rats. **(A)** One template was implanted randomly per subcutaneous pocket. In addition, to determine if the drug-eluting materials had systemic effects due to diffusion or convection, **(B)** SD templates with 0 mg/mL Cl-Am were implanted between SD templates with 5 mg/mL Cl-Am and **(C)** SD templates with 0 mg/mL Cl-Am were implanted alone.

Ten millimeter diameter discs of the templates (*n* = 3) were implanted on the back of Sprague-Dawley rats (300–325 g, male) following the protocol approved by the University of Memphis Institutional Animal Care and Use Committee as previously described (Fetz et al., 2017). Briefly, templates were disinfected with UV irradiation for 10 min on each side, and all materials were handled aseptically. One template lying flat was implanted per subcutaneous pocket, and the skin was closed with 2–3 staples. Four templates were implanted randomly on the back of each rat (Figure 1A). Additionally, to ensure drug-eluting materials exerted their effects locally without diffusion or convection to adjacent samples, two SD templates with 0 mg/mL Cl-Am were implanted between four SD templates with 5 mg/mL Cl-Am (*n* = 3), and two SD templates with 0 mg/mL Cl-Am (*n* = 3) were implanted alone (Figures 1B,C). One day after implantation, the animals were euthanized, the subcutaneous pockets were opened, and the templates were explanted, fixed, and stored in 10% neutral buffered formalin at 4°C.

Fixed templates were processed, sectioned at 10 μm, and stained with hematoxylin and eosin (H&E) following standard protocol. The sections were evaluated and scored by a board-certified veterinary pathologist blinded to sample identity for presence of surface DNA, invasion into the template, and degree of neutrophil degeneration (Table 2). The presence of surface DNA was evaluated based on prevalence of DNA on the surfaces of the templates seen as a blue-stained acellular layer, invasion into the templates was scored based on number of invading neutrophils and depth of invasion, and the degree of neutrophil degeneration was assessed based on the abundance of neutrophils showing degenerative morphological changes (i.e., loss of lobulated nuclei, fragmentation, apoptotic bodies, etc.). Finally, the surface of the 1 day templates were evaluated for template-bound CitH3 through an On-cell Western blot following the *in vitro* protocol.

**Table 2:** Electrospun templates were evaluated by a veterinary pathologist in a blinded fashion at 1 day after implantation in a subcutaneous rat implant model.

**Score**	**Presence of surface DNA**	**Invasion into the template**	**Degree of neutrophil degeneration**
1	DNA adherent to some surfaces of the template	Slight invasion into the template near a fold or defect in the template	Most invading neutrophils non-degenerative
2	DNA adherent to most surfaces of the template	Slight invasion into the template	Many invading neutrophils non-degenerative
3	Dense DNA layer adherent to some surfaces of the template	Moderate invasion with reduced invasion toward the center of the template	Occasional invading neutrophils non-degenerative
4	Dense DNA layer adherent to most surfaces of the template	Moderate invasion throughout the thickness of the membrane	Few non-degenerating neutrophils not deeply invaded
5	Conspicuous, dense DNA layer adherent to the surfaces of the template	Dense invasion throughout the thickness of the membrane	Diffuse degenerating neutrophils near the surface of defect in the template.

### Statistical analysis

Significant differences in Cl-Am activity were determined by an unpaired *t*-test to compare activity for each concentration. The plot of average fluorescent intensity against Cl-Am concentration was fit with a four-parameter logistic regression, and a lack of fit test was executed. The plot of relative fluorescence against mass of CitH3 was fit with a linear regression. For all other *in vitro* methods, analysis of data was conducted using a two-way analysis of variance (ANOVA) and a Tukey multiple comparison procedure. All *in vitro* statistical analyses were performed in GraphPad Prism 6 at a significance level of 0.05.

For the *in vivo* methods, the data sets were resampled (*n* = 1,000) using bootstrapping and then evaluated with a two-way ANOVA and a Tukey multiple comparison procedure to ensure the data did not violate the assumptions of normality (Erceg-Hurn and Mirosevich, 2008; Xu et al., 2013). Resampling and analyses were performed in SAS 9.4 and GraphPad Prism 6, respectively, at a significance level of 0.05.

## Results

### Cl-Am retains activity during template production

**Figure 2:**
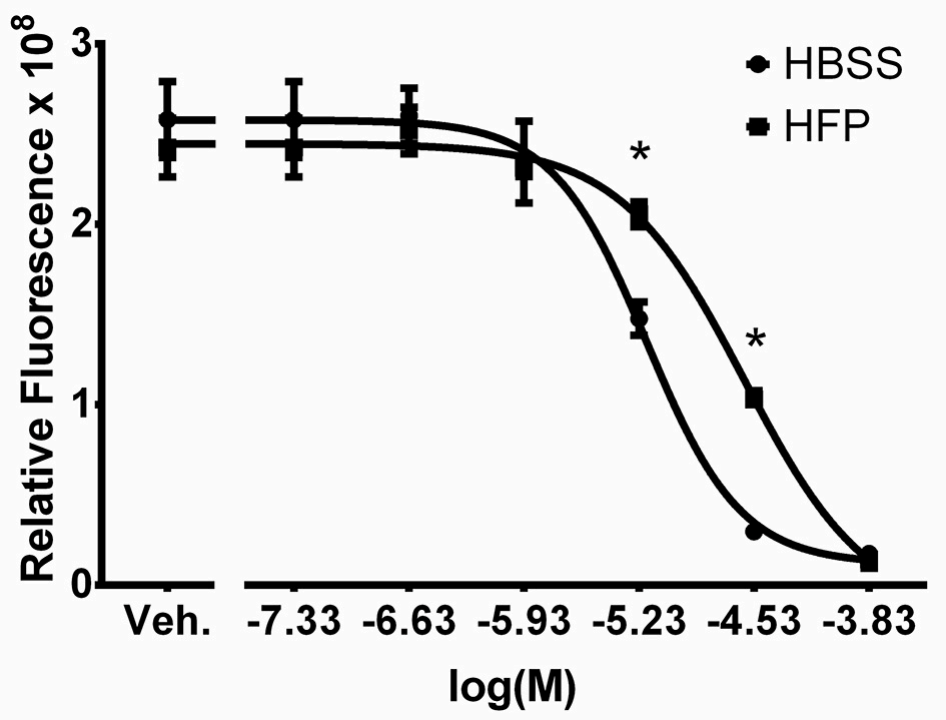
Cl-Am retains activity after exposure to HFP. Plot of drug concentration against relative fluorescence for HBSS and HFP exposed samples. Vehicle (Veh.) is HBSS only. The four-parameter regression of the HFP exposed samples has no evidence of lack of fit (*p* = 0.28). ^*^Significant difference in relative fluorescence (*p* < 0.05) (mean ± std. dev., *n* = 4).

Before incorporating the drug into the electrospun templates, the activity of Cl-Am was verified after exposure to the harsh organic solvent, HFP, that is used during electrospinning. Figure 2 shows the four-parameter regression curve for the HFP samples along with the HBSS samples. As the concentration of drug increases, the relative fluorescence decreases, signifying an increase in the inhibition of PAD4. The assayed drug concentrations were selected to provide a range that includes the IC50 of Cl-Am against PAD4, which is 5.9 μM in an *in vitro* model (Luo et al., 2006). Exposure of Cl-Am to HFP significantly reduced its activity against PAD4 at two concentrations, 5.9 and 29.5 μM (*p* < 0.0001). However, the drug was still active producing a sigmoidal curve similar to that of the HBSS samples. This loss of Cl-Am activity upon exposure to HFP was reproducible and therefore could be compensated for by increasing the drug concentration. Nonetheless, the drug retained efficacy after exposure to HFP and inhibited PAD4 activity at concentrations as low as 1.2 μM, demonstrating the potential to incorporate it into electrospun templates for release and activity against PAD4.

In addition to determining the activity of Cl-Am, the samples exposed to HFP were used to create a standard curve for quantifying unknown Cl-Am concentrations. The four-parameter logistic regression of the HFP exposed samples (Equation 1, *x* = logarithmic transform of Cl-Am concentration and *y* = relative fluorescence) has an *R*^2^-value of 0.996, and there is no evidence of an inadequate model from the lack of fit test (*p* = 0.28).

(1) $$\begin{array}{l} y=\;\frac{2.65}{1+10^{5.25+1.14x}} \end{array}$$

### Template characteristics incorporating Cl-Am

**Figure 3:**
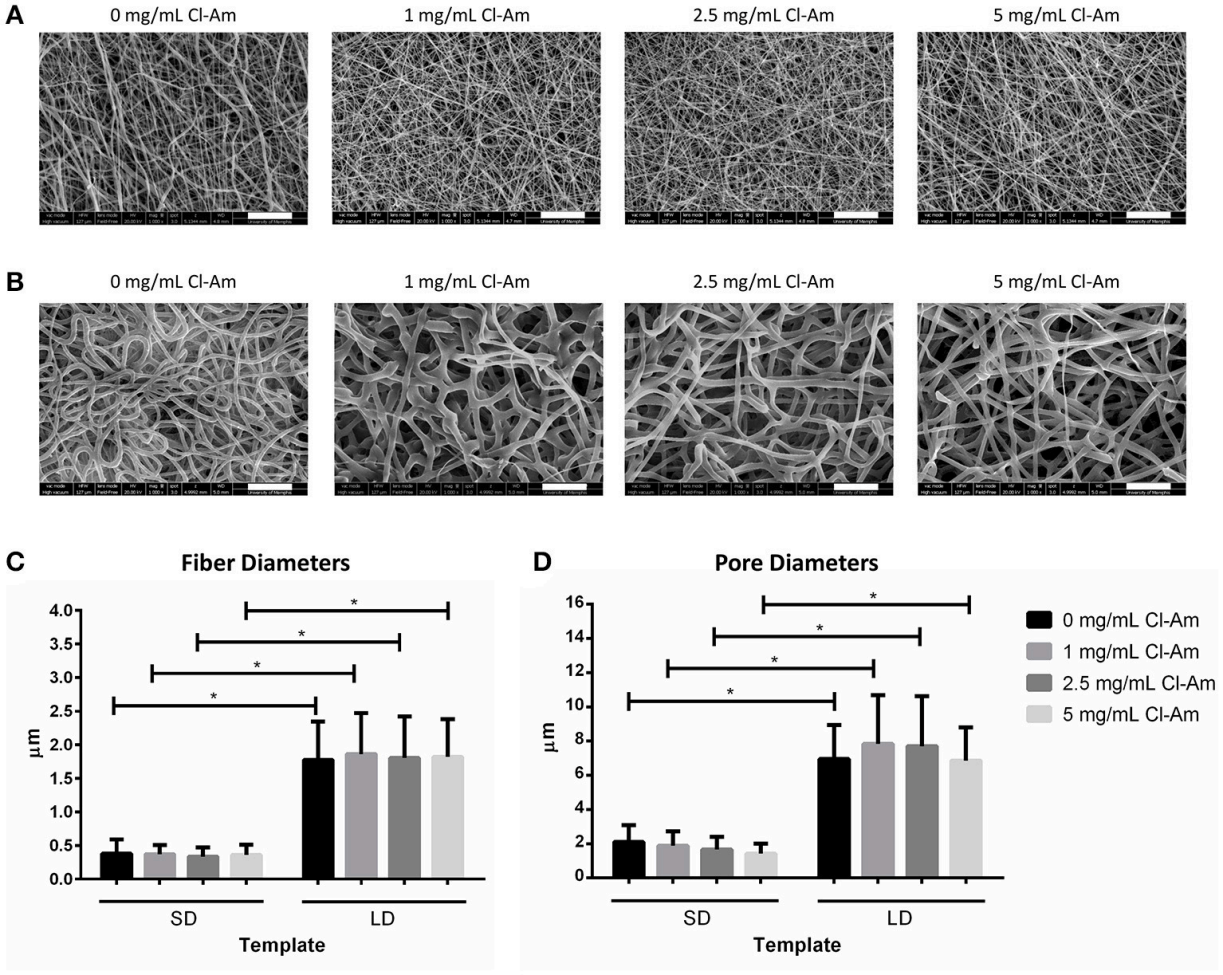
PDO electrospins at low and high concentrations with Cl-Am. Representative SEMs of **(A)** SD and **(B)** LD templates with 0–5 mg/mL Cl-Am (scale bars = 20 μm). For all drug concentrations, all SD and all LD templates have statistically equivalent **(C)** fiber and **(D)** pore diameters; however, at each drug concentration, SD templates have significantly smaller **(C)** fiber and **(D)** pore diameters than the large diameter templates. ^*^Significant difference (*p* < 0.05). (mean ± std. dev., *n* > 250 for fiber diameter analysis and *n* = 60 for pore diameter analysis).

Low and high polymer concentration solutions incorporating Cl-Am resulted in two distinct templates groups, SD and LD templates, which had uniform fiber and pore morphologies comparable to SD and LD templates without drug (Figures 3A,B). During electrospinning, the addition of a charged entity to the solution reduces fiber diameter, so we increased polymer concentration to prevent reduction in fiber diameter for Cl-Am templates (Lin et al., 2007). Figures 3C,D show the results of the fiber and pore diameter analyses, respectively. Within the SD and LD template groups, the templates did not have significantly different fiber diameters between 0 and 5 mg/mL Cl-Am. At each drug concentration, the SD templates had significantly smaller fiber diameters with an average of 0.36 ± 0.16 μm compared to the LD templates with average fiber diameters of 1.82 ± 0.59 μm (*p* < 0.05). Similarly, the pore diameters of the SD and LD templates were not different between templates with 0–5 mg/mL Cl-Am, but at each drug concentration, the SD templates had significantly smaller pore diameters near 1.76 ± 0.79 μm compared to the LD templates with average pore diameters of 7.34 ± 2.42 μm. Together, these data indicate that the increase in polymer concentration for SD and LD templates compensated for the addition of Cl-Am to produce templates with consistent fiber and pore diameters.

### Elution kinetics of Cl-Am from templates

**Figure 4:**
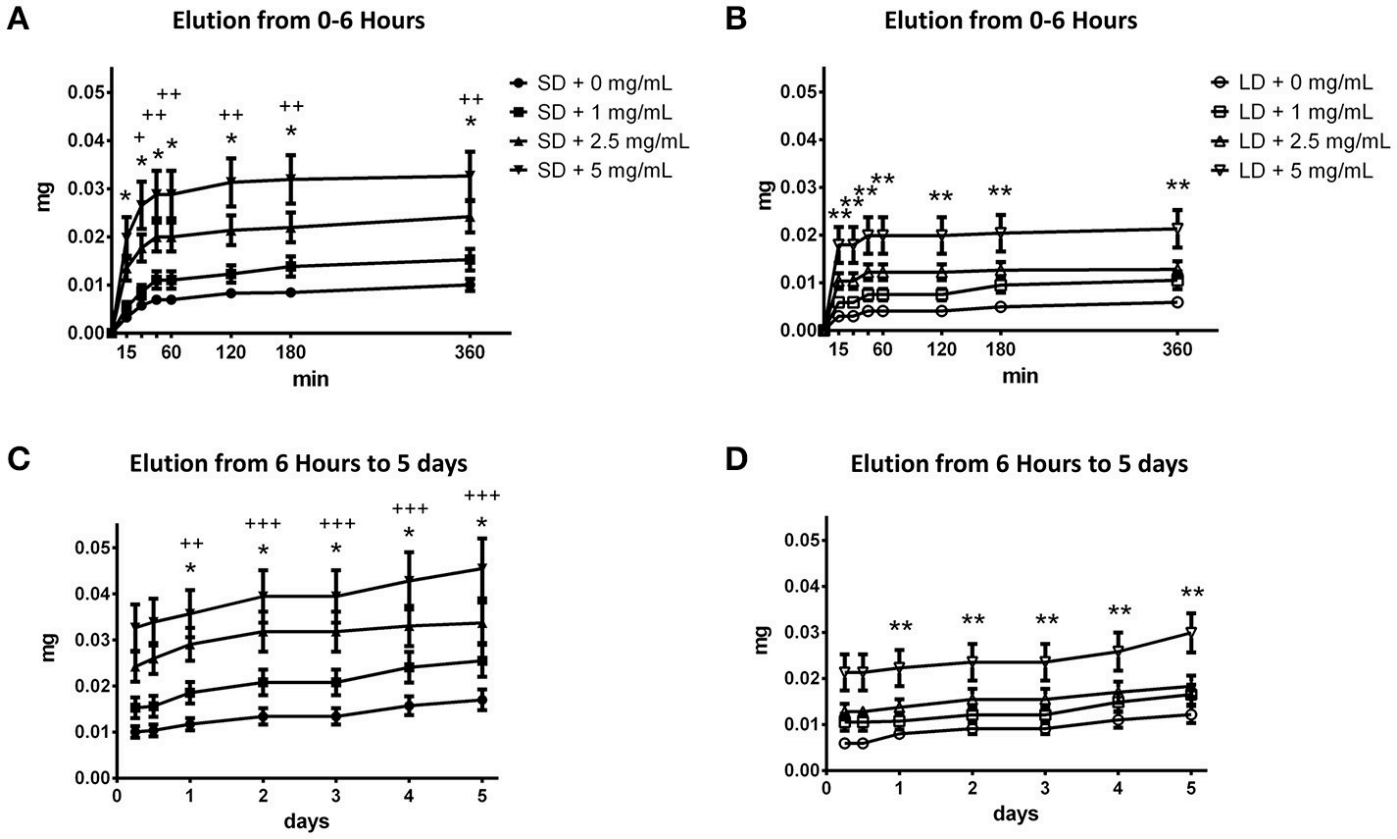
SD and LD templates elute Cl-Am over 5 days. From 0 min to 6 h, the **(A)** SD templates eluted significantly more drug than the **(B)** LD templates. This trend was observed from 6 h to 5 days for the **(C)** SD and **(D)** LD templates. ^*^Significant difference between 2.5 mg/mL Cl-Am and 5 mg/mL Cl-Am from 0 mg/mL Cl-Am templates (*p* < 0.05). ^**^Significant difference between 5 mg/mL Cl-Am from 0 mg/mL Cl-Am templates (*p* < 0.05). ^+^Significant difference between SD and LD templates with 5 mg/mL Cl-Am (*p* < 0.05). ^++^Significant difference between SD and LD templates with 2.5 mg/mL and 5 mg/mL Cl-Am (*p* < 0.05). ^+++^Significant difference between all SD and LD templates (*p* < 0.05) (mean ± std. dev., *n* = 6).

The electrospun templates eluted Cl-Am to varying degrees based on fiber diameter and drug content over 5 days (Figures 4A–D). At 30 min, the SD template with 5 mg/mL Cl-Am eluted significantly more drug than the LD template, and from 45 min to 6 h, the SD templates with 2.5 and 5 mg/mL Cl-Am eluted more drug than the LD templates (*p* < 0.05) (Figures 4A,B). These significant trends were seen over the 5-day period (Figures 4C,D), and by 2 days, all of the SD templates eluted significantly more Cl-Am than the LD templates (*p* < 0.05). Because HFP reduces the activity of Cl-Am, there is potential that some Cl-Am was eluted from the fibers and not detected in the activity assay. In addition, it is likely that some Cl-Am was retained within the fibers and had yet to elute from the templates in the 5-day period. The potential effects of low-level elution from the templates after the initial critical hours is negligible due to the short half-life of Cl-Am *in vivo* (Luo et al., 2006).

At 3 h, the SD templates with 1, 2.5, and 5 mg/mL Cl-Am had eluted the drug to reach concentrations of 108.7 ± 16.5, 172.4 ± 24.2, and 250.6 ± 39.5 μM Cl-Am. Contrastingly, the LD templates with 1, 2.5, and 5 mg/mL Cl-Am only eluted the drug to reach concentrations 74.4 ± 12.2, 99.2 ± 13.3, and 160.1 ± 30.2 μM Cl-Am. Despite the equivalent drug content during fabrication, these differences result from the high surface area to volume ratio of the SD templates, leading to a more significant burst release of Cl-Am. Because the SD templates elicit the highest degree of NETosis, the greater elution of Cl-Am from the SD templates at 3 h may counteract template-induced NETosis (Fetz et al., 2017). Furthermore, by 3 h, the SD and LD templates eluted nearly 60% of the drug released over the 5-day period. This burst release is due to the characteristic segregation of the charged drug to the outer surface of the electrospun fibers (Sun et al., 2008). Since the swarming of neutrophils to the site of inflammation occurs in the first 3 h, the substantial local release in the initial hours may be desired to regulate the critical interaction that directs the ensuing response (Lämmermann et al., 2013).

### Templates eluting Cl-Am modulate NETosis *in vitro*

**Figure 5:**
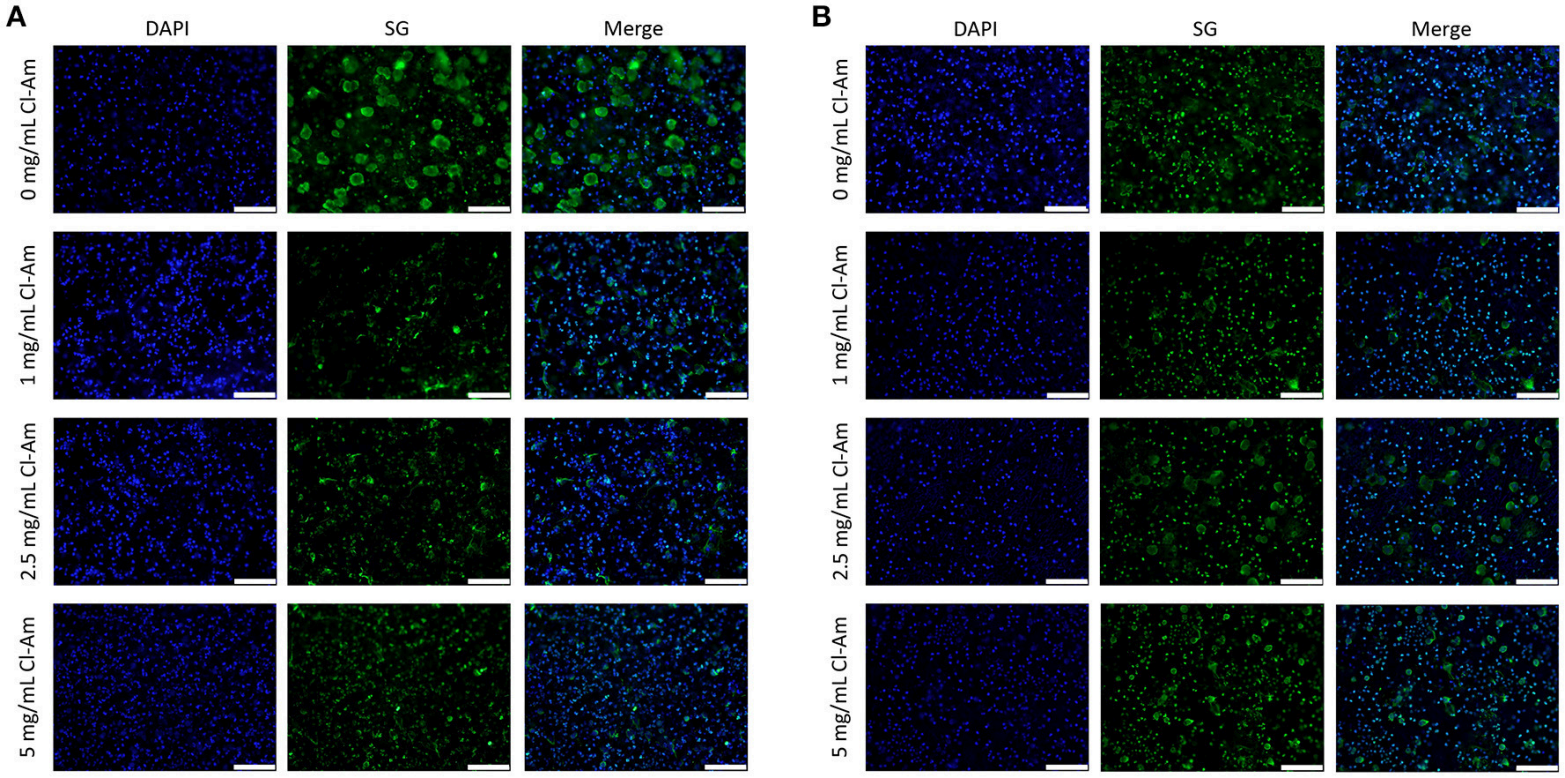
Neutrophils interacting with Cl-Am eluting templates exhibit different degrees of NETosis at 3 h. Representative fluorescent micrographs of **(A)** SD and **(B)** LD templates indicate different degrees of NETosis based on drug content (scale bars = 100 μm). Blue is DAPI. Green is SG.

**Figure 6:**
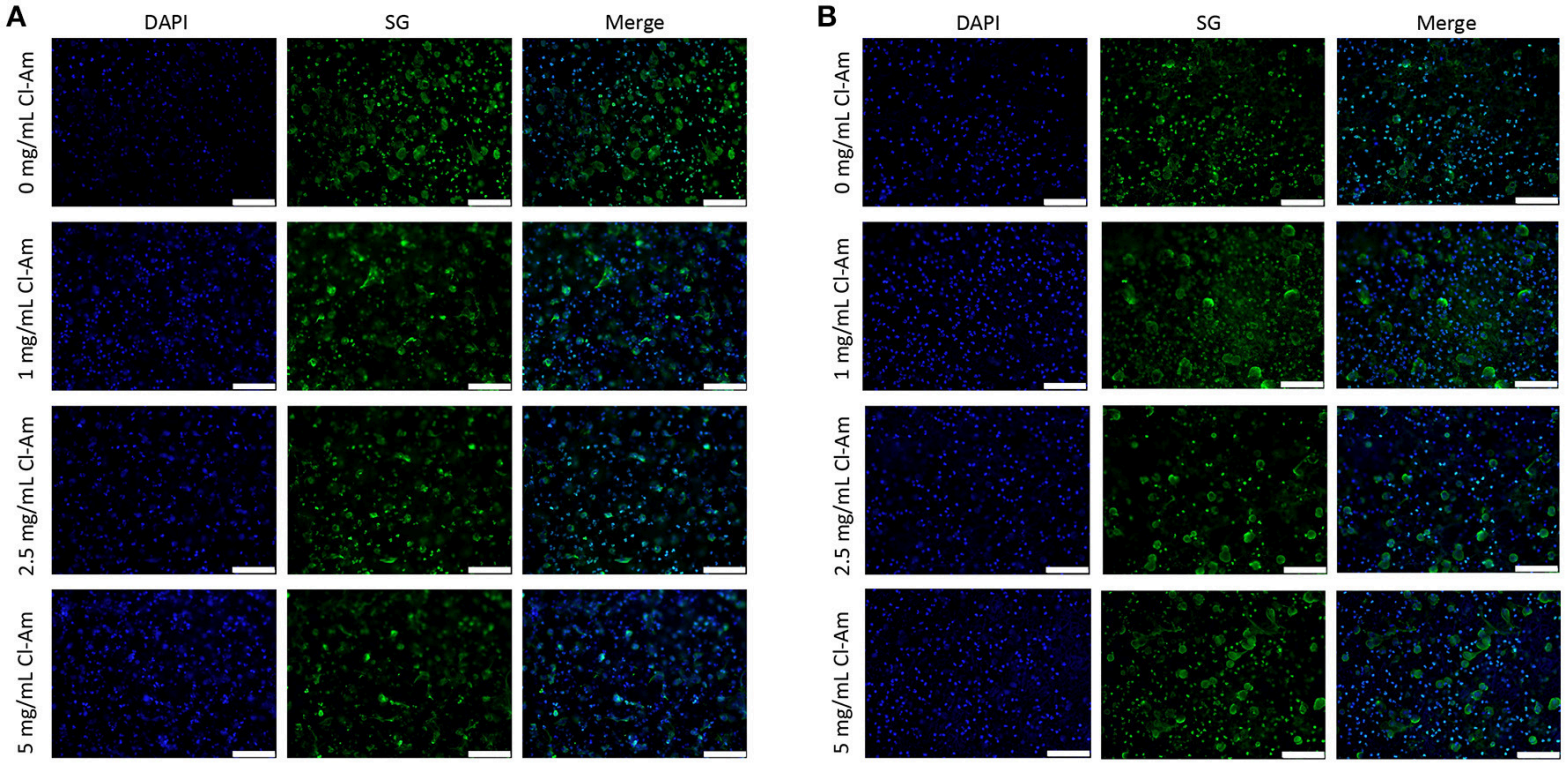
Neutrophils interacting with Cl-Am eluting templates exhibit equivalent degrees of NETosis at 6 h. Representative fluorescent micrographs of **(A)** SD and **(B)** LD templates indicate near equivalent degrees of NETosis based on drug content (scale bars = 100 μm). Blue is DAPI. Green is SG.

**Figure 7:**
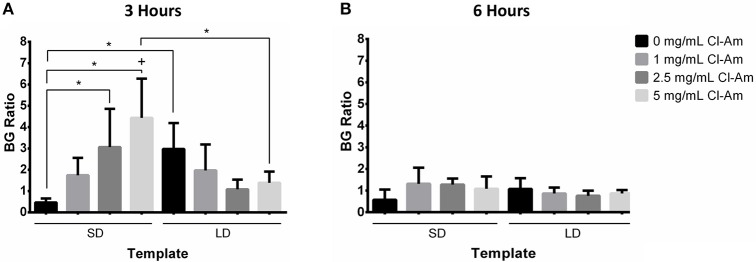
Cl-Am significantly decreases NETosis on SD templates. The BG ratio for templates at **(A)** 3 and **(B)** 6 h indicate that template architecture modulates NETosis. In addition, the elution of Cl-Am significantly affects NETosis at 3 h with minimal effects at 6 h. ^*^Significant difference (*p* < 0.05). ^+^Significant difference between 3 and 6 h (*p* < 0.05) (mean ± std. dev., *n* = 4).

Fluorescence microscopy revealed contrasting degrees of NETosis in response to the different template fiber diameters and drug concentrations at 3 and 6 h (Figures 5A,B, 6A,B). Blue (DAPI) stains condensed chromatin, and green (SG) stains decondensed NET chromatin, respectively. SG cannot penetrate an intact cell membrane (Lebaron et al., 1998); therefore, cyan, an overlap of DAPI and SG staining, indicates cells with an intact nucleus but compromised cell membrane. Because the cells retain their plasma membrane integrity while the nuclear and granular membranes disintegrate, these cyan stained neutrophils have compromised cell membranes and may be undergoing cell death other than NETosis. The varying degrees of NETosis were quantified with a BG ratio using a MATLAB image analysis algorithm (Figures 7A,B). A BG ratio >1 indicates that more image area of the template is covered by intact cells than extruded NETs, signifying a low degree of NETosis, with the inverse for a BG ratio <1, signifying a high degree of NETosis.

At 3 h, the SD templates with 0 mg/mL Cl-Am elicited a statistically higher degree of NETosis, with a BG ratio of 0.47 ± 0.19, than the LD template with 0 mg/mL Cl-Am, which had a BG ratio of 2.98 ± 1.21 (*p* < 0.05). These results were anticipated and replicate, to the same magnitude, those of our previous experiments where we showed that SD PDO templates increase NETosis *in vitro* while LD PDO templates led to a significant reduction (Fetz et al., 2017). As seen in Figure 7A, with increasing Cl-Am concentration, the degree of NETosis in response to the SD templates significantly decreases in a dose-dependent manner. The SD templates with 5 mg/mL Cl-Am have a BG ratio of 4.4 ± 1.84, nearly 10 times greater than the SD templates without drug (*p* < 0.05). These results suggest that the addition of Cl-Am to the SD templates attenuates NETosis through the inhibition of PAD4, and that these effects are concentration dependent.

For the LD templates at 3 h, the incorporation of Cl-Am into the templates decreases the BG ratio in a dose-dependent manner down to 1.39 ± 0.52 for the templates with 5 mg/mL Cl-Am. While not significant, this trend is opposite of the SD templates and suggests that in the microenvironment created by the LD templates with Cl-Am, NETosis is increased. Nonetheless, the BG ratio for all of the LD templates with Cl-Am is >1, indicating that there is still more surface area on the template covered by intact neutrophils than extruded NETs. Taken together, these contrasting effects of Cl-Am at 3 h *in vitro* suggest that there may be another mechanism in addition to PAD4-regulated NETosis that modulates the generation of NETs based on the template architecture. By 6 h, the BG ratios for the SD and LD templates decreased to values around 1, suggesting that there is an equivalent amount of area covered by viable neutrophils and extruded NETs on the templates (Figure 7B).

As a concomitant method to quantify NETosis, an On-cell Western assay was used to quantify template-bound CitH3 at 3 and 6 h *in vitro* (Figures 7A,B). Histone H3 is citrullinated by PAD4 during NETosis, generating CitH3, which is then complexed with released NETs (Neeli et al., 2008). Previously, we showed that detection of CitH3 can be used in addition to fluorescence microscopy to quantify the degree of NETosis in response to electrospun templates (Fetz et al., 2017). The linear regression of mass of CitH3 against relative fluorescence (Equation 2, *x* = mass in ng of CitH3 and *y* = infrared intensity) has an *R*^2^-value of 0.966, which indicates a well-fitting line, and linear relationship that can be used to quantify CitH3.

(2) $$\begin{array}{l} y=91.1x \end{array}$$

**Figure 8:**
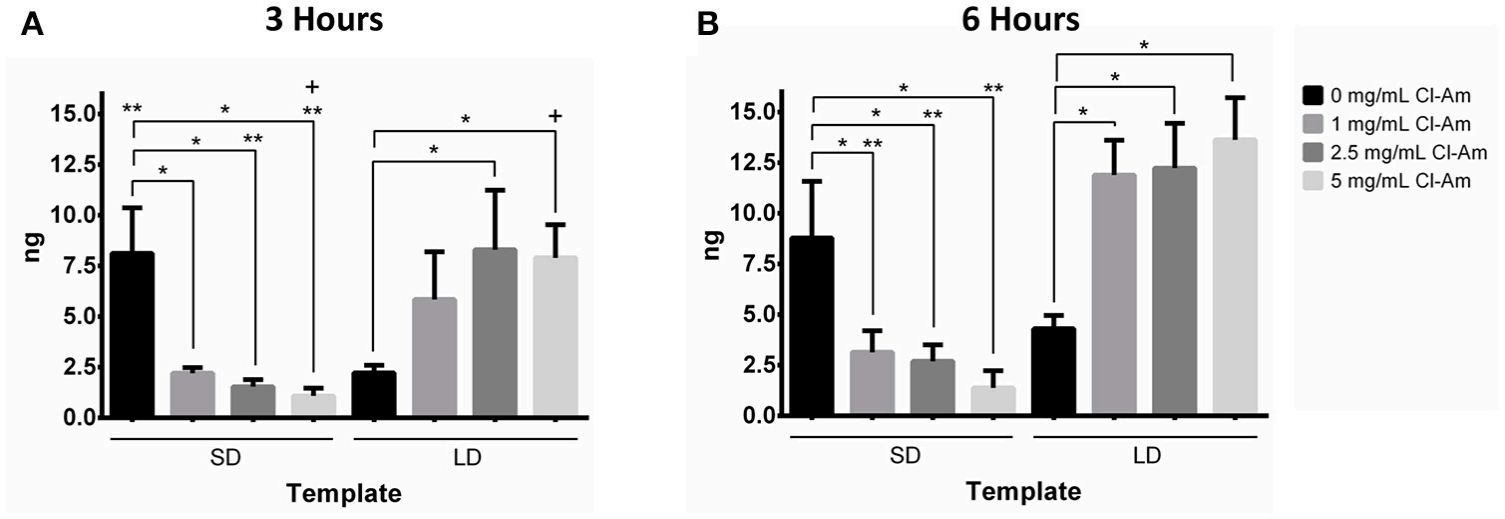
Cl-Am significantly decreases template-bound CitH3 on SD templates. Template-bound CitH3 at **(A)** 3 and **(B)** 6 h suggests that template architecture and Cl-Am concentration regulate NETosis. ^*^Significant difference (*p* < 0.05). ^**^Significant difference between SD and LD templates (*p* < 0.05). ^+^Significant difference between 3 and 6 h (*p* < 0.05) (mean ± std. dev., *n* = 4).

At 3 h, SD templates with 0 mg/mL Cl-Am had significantly more template-bound CitH3 than the LD templates with 0 mg/mL Cl-Am (*p* < 0.05). The SD templates had 8.1 ± 2.2 ng CitH3, nearly 4 times greater than the LD templates with 2.2 ± 0.4 ng. These results suggest a high degree of NETosis on the SD templates, which is supported by the results from fluorescence microscopy. In Figure 8A, Cl-Am significantly decreased the amount of detectable CitH3 on SD templates, which suggests a decrease in NETosis similar to the results from fluorescence microscopy (*p* < 0.05). Likewise, for the LD templates, the templates with Cl-Am correlated to higher amounts of template-bound CitH3. All of the LD templates with drug had more detectable CitH3 than their SD template counterparts. These similar results were also observed at 6 h (Figure 8B). However, while it increased on the LD templates, the amount of template-bound CitH3 did not increase on the SD templates with Cl-Am. Clearly, the elution of Cl-Am from the electrospun PDO templates regulates NETosis from the interacting neutrophils in a dynamic manner dependent on the template microenvironment.

### PDO templates eluting Cl-Am modulate NETosis *in vivo*

**Figure 9:**
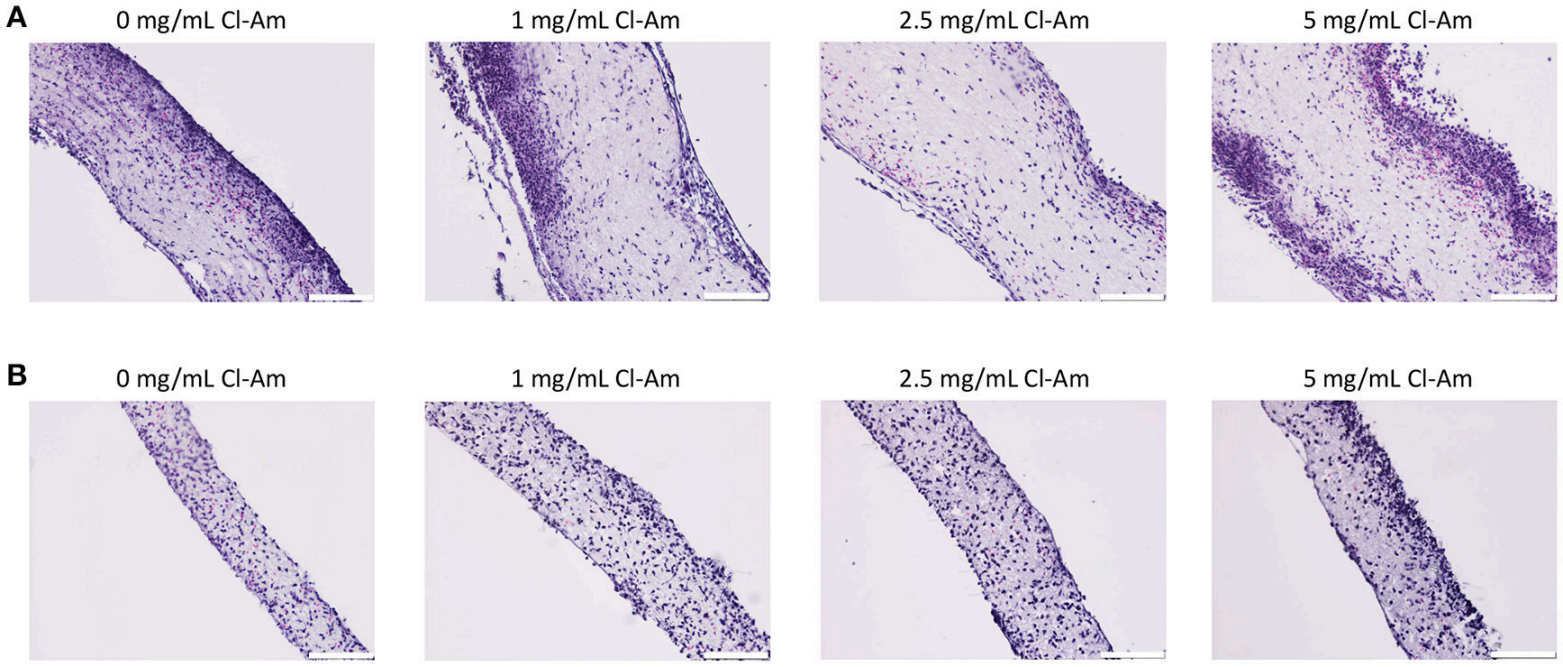
Cl-Am eluting electrospun templates modulate neutrophil behavior *in vivo*. Representative light microscopy images of H&E stained sections of **(A)** SD and **(B)** LD templates removed after 1 day (scale bars = 100 μm).

**Figure 10:**
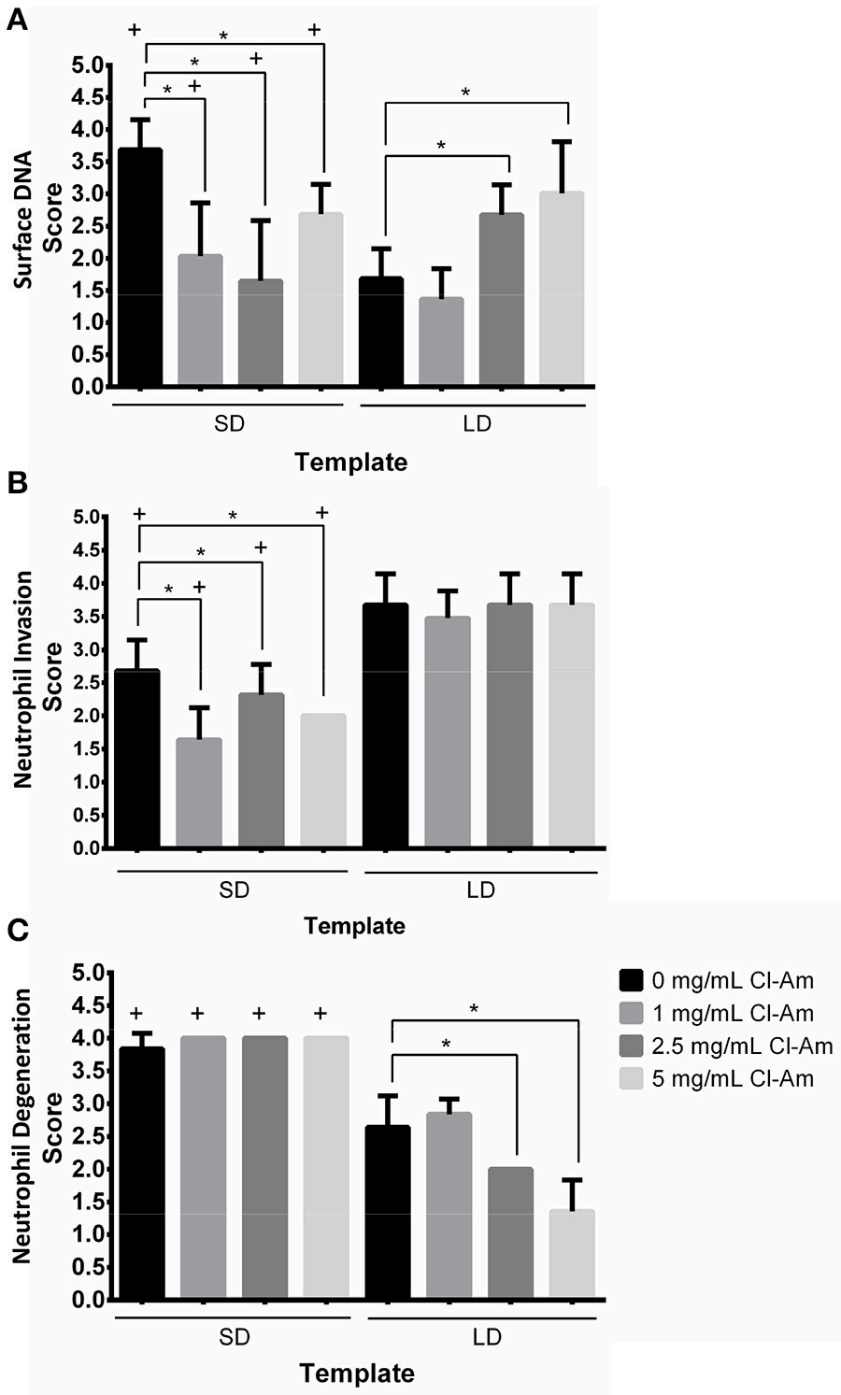
Cl-Am eluting templates modulate cell behavior *in vivo*. Histological scores obtained from a blinded, veterinary pathologist for **(A)** presence of surface DNA, **(B)** invasion into the template, and **(C)** degree of neutrophil degeneration after 1 day implantation. ^*^Significant difference (*p* < 0.05). ^+^Significant difference between SD and LD templates (*p* < 0.05) (mean ± std. dev., *n* = 3).

Next, the Cl-Am eluting electrospun templates were implanted subcutaneously on the back of rats. H&E staining of templates after 1 day (Figures 9A,B) indicated that SD and LD templates initiated differing responses based on template architecture and drug content, which were quantified by a veterinary pathologist in a blinded fashion. At 1 day, the presence of surface DNA, invasion into the template, and degree of neutrophil degeneration were evaluated (Figures 10A–C). The presence of surface DNA (Figure 10A) scored significantly higher on SD templates with 0 mg/mL Cl-Am at 3.7 ± 0.5 compared to LD templates with 0 mg/mL Cl-Am at 1.7 ± 0.5 (*p* < 0.05). A dense DNA layer was adherent to most surfaces on the SD the templates whereas only some DNA was adherent to the LD template surfaces, similar to the trends seen *in vitro*. For the SD templates, Cl-Am significantly decreased the scores for the presence of surface DNA, while for LD templates, the opposite was observed (*p* < 0.05). Again, these data reflect the fluorescence microscopy results *in vitro*, suggesting that Cl-Am elution results in similar effects in a physiological environment. Interestingly, for the SD templates with 5 mg/mL Cl-Am, the score for surface DNA was greater than the score for 1 and 2.5 mg/mL Cl-Am; however, the score was still significantly lower compared to SD templates with 0 mg/mL Cl-Am.

Invasion of neutrophils into the templates (Figure 10B) was also modulated by architecture and drug concentration. LD templates scored significantly higher with moderate invasion throughout the thickness of the template compared to SD templates with slight invasion into the template (*p* < 0.05). Because of the restrictive pore diameters of the SD templates, which are 3 times smaller than the LD template pore diameters, it is not surprising that the SD templates exhibited less invasion. Interestingly, the elution of Cl-Am from SD templates significantly decreased invasion into the templates compared to the SD template with 0 mg/mL Cl-Am (*p* < 0.05). This is not observed for the LD templates, which all scored with moderate invasion.

The degree of neutrophil degeneration (Figure 10C) was significantly different in response to the templates after acute interaction *in vivo*. As noted, the degeneration of neutrophils was classified by the degree of morphological changes. All of the SD templates scored significantly higher at 4.0 ± 0.2 compared to LD templates at or below 2.6 ± 0.5 (*p* < 0.05). This equates to minimal non-degenerating neutrophils that are not deeply invaded and many invading, non-degenerative neutrophils, respectively. Importantly, with increasing Cl-Am concentration, the LD templates scored significantly better for neutrophil degeneration, decreasing from 2.6 ± 0.5 for 0 mg/mL Cl-Am to 1.3 ± 0.5 for 5 mg/mL Cl-Am (*p* < 0.05). The decreasing scores suggest that with increasing Cl-Am concentration, a greater portion of the interacting neutrophils are viable and exerting their potential long-term effector functions to modulate the early-stage innate immune response.

**Figure 11:**
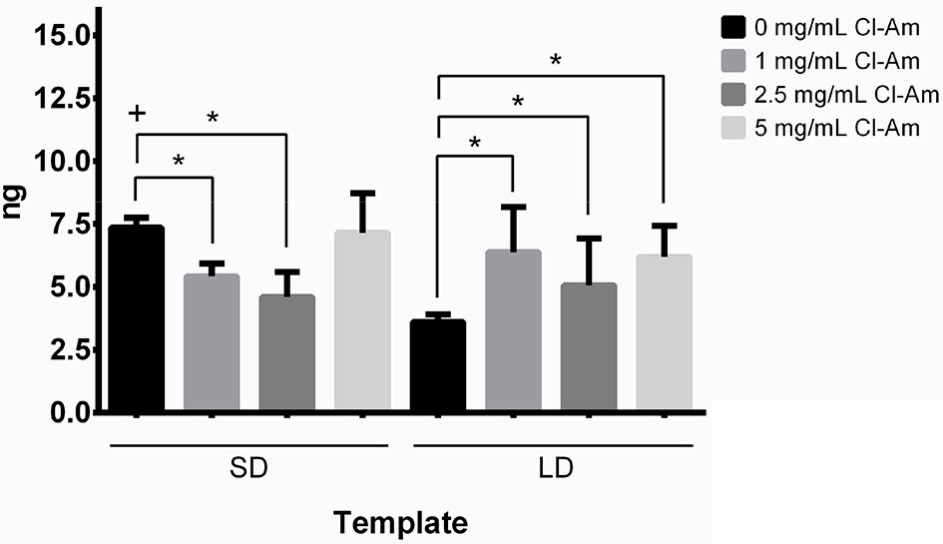
PDO templates eluting Cl-Am modulate NETosis *in vivo*. Cl-Am attenuates NETosis as indicated by CitH3 on SD templates while increasing NETosis on LD templates. ^*^Significant difference (*p* < 0.05). ^+^Significant difference between SD and LD templates (*p* < 0.05) (mean ± std. dev., *n* = 3).

In addition to pathological evaluation, the templates excised after 1 day were evaluated for template-bound CitH3 (Figure 11). The results show that the SD templates with 0 mg/mL Cl-Am had significantly more template-bound CitH3 with 7.3 ± 0.4 ng compared to the LD templates with 0 mg/mL Cl-Am at 3.6 ± 0.3 ng (*p* < 0.05). With increasing drug concentration, the amount of template-bound CitH3 significantly decreased on the SD templates, while the opposite was observed on LD templates (*p* < 0.05). However, the SD templates with 5 mg/mL Cl-Am resulted in more CitH3 compared to 1 and 2.5 mg/mL Cl-Am. These significant trends validate the scores categorized by the pathologist for presence of surface DNA on the templates, indicating that the Cl-Am eluting templates modulate NETosis *in vivo*.

To ensure the Cl-Am eluting templates exhibited only local inhibition of PAD4, SD templates with 0 mg/mL Cl-Am were implanted between templates with 5 mg/mL Cl-Am (Figure 1B). SD templates with 0 and 5 mg/mL Cl-Am were selected to maximally challenge the effects of Cl-Am through its high and rapid release from the SD templates. The SD templates with 0 mg/mL Cl-Am adjacent to templates with 5 mg/mL Cl-Am were compared to SD templates with 0 mg/mL Cl-Am that were implanted alone (Figure 1C). After 1 day, histological scores were not significantly different for presence of surface DNA, invasion into the template, and degree of neutrophil degeneration for the SD adjacent templates and the SD template controls (*p* < 0.05). The presence of DNA scored 2.0 ± 0.8 and 2.3 ± 0.8, the invasion into the templates scored 3.0 ± 0.7 and 3.0 ± 0.9, and the degree of neutrophil degeneration scored 4.2 ± 0.4 and 4.2 ± 0.2 for the SD adjacent templates and the SD template controls, respectively. Together, these data verify that the Cl-Am eluting templates only exert local effects in the first 24 h *in vivo* and do not affect the response to adjacent materials through diffusion or local convection.

## Discussion

The unregulated release of NETs from the acute neutrophil response may inhibit biomaterial-guided tissue regeneration. Aggregated NETs have been shown to degrade the potent cytokines and chemokines secreted by neutrophils to direct wound healing, impairing the healing response and potential for tissue regeneration (Schauer et al., 2014). Moreover, NETs are linked to the formation of fibrotic tissue (Riehl et al., 2016). In particular, the up-regulation of NETosis contributes to the formation of dense, fibrotic tissue in pulmonary fibrosis, fibrosis after myocardial infarction, and atherosclerosis (Jones et al., 1998; Rainer et al., 2014; Marino et al., 2015). Riehl et al. linked NETs to fibrosis through NET-bound, histone-induced activation of platelets resulting in transforming growth factor β secretion and the differentiation and activation of myofibroblasts (Riehl et al., 2016). Indeed, our initial work evaluating the neutrophil response to electrospun templates indicates that abundant template-preconditioning NETs induce fibrotic tissue formation *in vivo* and impair tissue integration with the biomaterial (Fetz et al., 2017).

We attempted to modulate the degree of NETosis by incorporating Cl-Am into electrospun templates for local delivery into the template microenvironment. Cl-Am, which irreversibly inhibits PAD4, has a short half-life *in vivo* of <15 min and preferentially antagonizes the active form of PAD4 through covalent binding, which together reduces off target effects outside of an inflammatory environment (Luo et al., 2006; Lewis et al., 2015). We are not the first to use Cl-Am to inhibit NETosis through irreversible inhibition of PAD4-mediated histone H3 citrullination. Kusunoki et al. used intraperitoneal injections of Cl-Am to reduce NETosis in a mouse model of small-vessel vasculitis (Kusunoki et al., 2016). In another study, subcutaneous injections of Cl-Am were used to attenuate NETs in a model of obesity-associated, low-grade chronic inflammation (Braster et al., 2016). However, we are the first, to our knowledge, to incorporate it into a delivery vehicle for local elution into a biomaterial-induced, inflammatory microenvironment.

First, we demonstrated that Cl-Am can be incorporated into electrospun templates, creating templates with distinct architectures that have been shown to polarize macrophages and previously regulate NETosis (Garg et al., 2013; Fetz et al., 2017). Next, we showed that Cl-Am eluted from the templates rapidly, which is characteristic of electrospun materials incorporating charge carries (i.e., Cl-Am) that segregate to the outer surface of the fibers (Sun et al., 2008). The burst release is highly desired for this application because the swarming of neutrophils to a site of inflammation occurs in the first 3 h after injury. After 3 h, swarming has nearly ceased so that the neutrophils can begin exerting their effector functions (Lämmermann et al., 2013). Thus, our delivery vehicle ideally released most of its potent payload in the critical window of neutrophil-template interactions.

With freshly isolated human peripheral blood neutrophils, we evaluated the neutrophil interactions with the electrospun templates *in vitro* for 3 and 6 h. The LD templates with 0 mg/mL Cl-Am significantly attenuated NETosis compared to the SD templates, which was quantified with fluorescence microscopy and template-bound CitH3. As the concentration of Cl-Am increased in the SD templates, the degree of NETosis was significantly reduced in a dose-dependent manner. Intriguingly, the opposite effect was observed for the LD templates.

By 6 h *in vitro*, all of the SD and LD templates triggered nearly equivalent degrees of NETosis, regardless of drug content. Those templates that attenuated NETosis at 3 h were unable to inhibit it at 6 h, which may be an artifact of *in vitro* culture from decreased neutrophil viability. Interestingly, while the fluorescent quantification reflects the increase in NETs on SD templates with Cl-Am at 6 h, the infrared detection of template-bound CitH3 did not. Considering the increase in NETosis on LD templates with Cl-Am, these data suggest that template-induced NETosis may also be occurring independent of PAD4-mediated histone H3 deimination.

Three models of NETosis have been proposed: suicidal NETosis dependent on reactive oxygen species (ROS), vital NETosis independent of ROS, and vital NETosis dependent on ROS (Delgado-Rizo et al., 2017). While the mechanisms are not yet fully understood, suicidal NETosis depends on ROS for deimination of histone H3 by PAD4 (Lewis et al., 2015). This is the model that we speculated is involved in template-preconditioning NETosis. Contrastingly, both forms of vital NETosis can occur independent of histone citrullination (Yousefi et al., 2009; Pilsczek et al., 2010). Therefore, our data suggest that they may also be involved in the neutrophil response to PDO templates.

The different types of NETosis are in part regulated by receptor signaling through toll-like receptors, complement receptors, cytokine receptors, and integrins (Brinkmann et al., 2004; Munks et al., 2010; Garcia-Romo et al., 2011; Carestia et al., 2016). Because these same receptors interact with soluble proteins adsorbed on the template surface, variation in ligand binding and activation of outside-in signaling likely contributes to the activation of the different mechanisms triggering NETosis. We hypothesize that protein adsorption and the conformational changes regulate outside-in signaling for template-induced responses. Particularly, we anticipate greater protein adsorption on SD templates (i.e., high surface area to volume ratio) that is dynamic as described by the Vroman effect, and that exposure of protein domains through conformational changes induced by hydrophobic interactions engage different signaling pathways, topics of further investigation (Hirsh et al., 2013). This type of regulation provides an explanation for the contrasting affects of Cl-Am based on fiber diameter. The ability of Cl-Am to inhibit template-induced NETosis may be intimately linked to protein adsorption on the template and the engagement of neutrophil surface receptors, which will be addressed in future studies.

The results of the subcutaneous rat implant model were similar to the *in vitro* evaluation with freshly isolated human neutrophils and indicated dose-dependent trends modulating the early-stage innate immune response. With the addition of Cl-Am to the SD templates, the degree of NETosis significantly decreased, except for the SD templates with 5 mg/mL Cl-Am, and the opposite was observed for LD templates. We anticipated that the dose-dependent effects observed *in vitro* would translate to the *in vivo* model. Therefore, this interesting discrepancy for SD templates with 5 mg/mL Cl-Am between the *in vitro* and *in vivo* data suggests that the physiological complexity of the *in vivo* microenvironment masks the dose-dependent effects of Cl-Am for the SD templates.

Despite the increase in NETosis, all of the LD templates had significantly greater neutrophil invasion compared to the SD templates after 1 day *in vivo*. Invasion into the templates may be a critical driving force for tissue integration and regeneration. Neutrophils secrete potent pro-angiogenic factors in large quantities, like inhibitor free matrix metalloproteinase 9 (MMP-9), to guide and direct the growth of new blood vessels (Ardi et al., 2007). Without neutrophils, Lin et al. found vascularization of their biomaterial abrogated (Lin et al., 2017). Regulating NETosis and neutrophil invasion may work in tandem to enhance tissue integration and regeneration.

We previously suggested that the restrictive pores of the SD templates may be increasing NETosis due to the decreased invasion of neutrophils into the templates (Fetz et al., 2017). However, considering the histological scores for the presence of surface DNA and invasion, the restrictive pore diameters are not a factor that could be increasing NETosis with the LD templates incorporating Cl-Am. Since neutrophils have been shown to sense size and selectively release NETs, it may be the diameter of the fibers (i.e., micron-sized) that are up-regulating NETosis on the LD templates eluting Cl-Am (Branzk et al., 2014).

Importantly, the templates modulated acute neutrophil degeneration *in vivo* with all of the SD templates significantly increasing degeneration compared to LD templates. Degeneration was ranked based on the proportion of neutrophils showing degenerative signs such as loss of lobulated nuclei and fragmentation. Thus, the SD templates promote significant degeneration at 24 h *in vivo*. Because the scores for the presence of DNA improved with Cl-Am, these data suggest that the cells interacting with the SD templates may be dying through other forms of cell death in addition to NETosis, like rapid apoptosis or necrosis. Moreover, the degeneration scores for the LD templates eluting Cl-Am decreased significantly in a dose-dependent manner, despite an increase in the presence of surface DNA. Therefore, a greater portion of the neutrophils that are interacting with the template, invading it, and surviving are able to employ their long-term effector functions like the secretion MMP-9 for biomaterial-guided angiogenesis (Ardi et al., 2009). Taken together, these data suggest that engineering a Cl-Am delivery vehicle with the pore diameters of the LD templates and the fiber diameters of the SD templates may create the ideal microenvironment for regulated NETosis.

While PAD4 is a prominent therapeutic target that can be antagonized with Cl-Am to regulate NETosis, there are many mechanisms involved in the NETosis response to biomaterials that provide other targets for intervention (Radic and Neeli, 2013; Douda et al., 2015). Our preliminary evaluation suggests that other pathways may be regulating the release of NETs in the presence of Cl-Am and that the complexity of the physiological environment should be considered. The deimination of substrates by PAD4 may be linked to tissue necrosis factor alpha signaling, suggesting that synergistically targeting cytokine and chemokine production may further modulate NETosis (Mastronardi et al., 2006). Another recent study identified that PAD4 inhibition could impact neutrophil cytokine secretion, which may impact neutrophil polarization and the release of innate immune regulators (Sun et al., 2017). Future work should examine how fiber and pore diameters regulate NETosis independently, other mechanisms that may be regulating NETosis in the template microenvironment, and how Cl-Am elution effects neutrophil polarization. Extended *in vivo* studies should also be performed to understand the long-term effects of PAD4 inhibition by Cl-Am in tissue regeneration. Clearly, local delivery of Cl-Am modulates neutrophil NETosis *in vitro* and *in vivo* and may have significant implications in biomaterial-guided tissue regeneration and other medical devices that fail through fibrotic encapsulation.

## Conclusion

Our preliminary evaluation of Cl-Am eluting, electrospun PDO templates demonstrates that Cl-Am can be used to modulate NETosis in response to a biomaterial, thereby regulating acute inflammation and the early innate immune response. We demonstrated the efficacy of using electrospun templates to deliver Cl-Am locally as well as its ability to regulate NETosis in a dose-dependent manner *in vitro* with fresh human peripheral blood neutrophils and *in vivo* with a rat subcutaneous implant model. It is evident that the innate immune response is highly complex and much remains to characterize. However, we have shown the significance of designing immunomodulatory biomaterials that regulate the neutrophil interaction to potentially improve tissue integration and regeneration. Further investigation of the neutrophil response to biomaterials and the therapeutic potential of Cl-Am is needed and will likely yield significant insight in the field of tissue engineering and regenerative medicine.

## Author contributions

AF: executed all *in vitro* and *in vivo* experiments, conducted all data analyses, and composed the manuscript; IN: facilitated the isolation of human neutrophils and assisted with *in vitro* culture of neutrophils; KB: performed the animal surgeries; RR: conducted histological evaluation and scoring; MS: provided guidance on data analysis methods; MR: provided input on experimental design and manuscript preparation; GB: provided input on experimental design and manuscript preparation. All authors reviewed and commented on the manuscript.

### Conflict of interest statement

Author RR is employed by TriMetis Life Sciences. The rest of the authors declare that the research was conducted in the absence of any commercial or financial relationships that could be construed as a potential conflict of interest.
